# State-of-the-Art Review: Anatomical and Imaging Considerations During Transcatheter Tricuspid Valve Repair Using an Annuloplasty Approach

**DOI:** 10.3389/fcvm.2021.619605

**Published:** 2021-02-05

**Authors:** Nina C. Wunderlich, Melanie Landendinger, Martin Arnold, Stephan Achenbach, Martin J. Swaans, Robert J. Siegel, Siew Yen Ho

**Affiliations:** ^1^Cardiovasclar Center Darmstadt, Darmstadt, Germany; ^2^Department of Cardiology, Friedrich-Alexander-Universität Erlangen-Nürnberg, Erlangen, Germany; ^3^Department of Cardiology, St. Antonius Hospital, Nieuwegein, Netherlands; ^4^Cedars-Sinai Medical Center, Smidt Heart Institute, Los Angeles, CA, United States; ^5^Cardiac Morphology Unit, Royal Brompton Hospital, London, United Kingdom

**Keywords:** tricuspid valve, annuloplasty, transesophageal echocardiography, functional tricuspid regurgitation, transcatheter intervention

## Abstract

Transcatheter techniques for the treatment of tricuspid regurgitation (TR) are being more frequently used and several new devices are in development. Since 90% of patients with TR have secondary TR, catheter based systems which reduce the dilated tricuspid annulus area are of particular interest. In order to perform an annuloplasty procedure effectively and safely, knowledge about the anatomy of the tricuspid valve apparatus and especially of the annulus in relation to the important neighboring structures such as the aortic root, the RCA, the electrical pathways and the CS is fundamental. In addition, comprehensive understanding of the device itself, the delivery system, its maneuverability and the individual procedural steps is required. Furthermore, the use of multi-modality imaging is important. For each step of the procedure the appropriate imaging modality as well as the optimal; imaging planes are crucial to provide the necessary information to best guide the individual procedural step.

## Introduction

In recent years, interest in the transcatheter treatment of tricuspid regurgitation (TR) has considerably increased. TR (including all grades of severity) is a common valve disorder that affects 65–85% of adults (1, 2). In ~90% of all cases TR is functional, being secondary TR due to tricuspid annular dilation with or without leaflet tethering causing malcoaptation (3, 4). Most commonly this is a consequence of left-sided valvular or myocardial disease (>60% of cases) or associated with pulmonary hypertension. In ~10% of all patients with TR, TR is primary (organic) with an intrinsic abnormality of the tricuspid valve itself, which can be either congenital or acquired (3, 5). Primary TR occurs in association with Ebstein‘s anomaly, atrioventricular septal defects or with myxomatous valvular changes resulting in tricuspid valve prolapse. Acquired TR may be caused by endocarditis, rheumatic disease, carcinoid, infiltrative valvulopathy or by an iatrogenic trauma [e.g., by right ventricular (RV) pacemaker leads/RV myocardial biopsy (6–8)]. The prevalence of TR increases with age and is significantly more common in women (9). Mild TR is ubiquitous and not considered pathological as it does not cause RV dysfunction, RV dilation or worsen prognosis

(9). However, moderate or greater TR was identified as independently associated with increased mortality in a study that examined the influence of a TR on hospitalization for heart failure and mortality in 33,305 patients (10). The majority of patients with TR are treated medically, with drug therapy options essentially limited to diuretics and vasodilators in selected patients (11). In the United States only a small number of patients (<8,000 of 1.600.000 patients with moderate-to-severe TR) undergo tricuspid valve surgery (either valve repair or replacement) (12, 13), The low incidence of surgery is thought to be due to outcomes associated with surgical therapy of an isolated TR:

- The in-hospital mortality rate is currently 8.8% (14)
- Isolated tricuspid valve procedures carry the highest risk of all valve surgeries (15)
- Single center experiences with isolated tricuspid valve surgery are limited by small numbers (16)
- The length-of-stay is relatively long: median 11 days (associated with higher costs) (14)
- Pacemakers are utilized in a high number of patients undergoing TV replacement (26%) (14)
- Tricuspid valve replacement, age >60 years, end-stage renal disease and coagulopathies are associated with greater in-hospital mortality (14)

In accordance with current guidelines (11, 17), 86% of tricuspid valve surgeries are performed in patients with other surgical indications whereas surgeries in patients with isolated TR are less common (14%) (18). Mortality rates range between 2 and 10% after isolated tricuspid valve intervention. Prognostic data are limited and do not show improved survival after surgical tricuspid valve repair or replacement (19–21). Hence, new, less invasive therapies to treat TR are needed. A propensity-matched case-control study by Taramasso et al. (22) recently showed promising results. The authors demonstrated, that transcatheter tricuspid valve intervention using different devices and approaches is associated with improved survival and reduced heart failure rehospitalization compared to medical therapy (22).

Since secondary (functional) TR accounts for 90% of the cases in adults, annular dilation is a primary target for therapeutic transcatheter repair strategies. Consequently devices which reduce tricuspid annular dimensions are of interest.

In this review we focus on the annuloplasty approach using the Cardioband (Edwards Lifesciences, Irvine, CA, USA). The Cardioband is the only annuloplasty device with Conformitié Européenne (CE) mark approval. Results from the TRI-REPAIR (TrIcuspid Regurgitation RePAIr With CaRdioband Transcatheter System) study demonstrated a significant reduction in TR by reducing annular dimensions [the effective regurgitant orifice area was reduced by 50% (0.8 vs. 0.4 cm^2^; *p* < 0.01), and the mean vena contracta width by 28% (1.2 vs. 0.9 cm; *p* < 0.01)] with improvement in heart failure symptoms, quality of life and exercise tolerance after 6 months (23).

In this overview we describe important anatomical structures and anatomical relationships that are relevant for the procedure and a practical guide to peri- procedural imaging according to our initial experiences with the Cardioband device.

## Anatomy of the Tricuspid Valve and Relationships to Surrounding Structures

The tricuspid valve is a complex of interconnected components that includes leaflets hinged at the atrioventricular junction and suspended by tendinous chords (chordae tendinea) attached to the ventricular septum or to papillary muscles that, in turn arise from the ventricular wall. At the hinge of the leaflets, atrial myocardium may overlap the leaflet surface by 0.5–2 mm. Thus, normal valvular function requires not only normality of all the valvular components but also adjoining atrial and ventricular walls for sphincteric contraction and excursion of the orifice toward the ventricular apex, as well as interaction with the left ventricle through muscular continuity.

### Tricuspid “Annulus” or Atrioventricular Junction?

It is underappreciated that the tricuspid annulus is not a robust fibrous ring which suspends the leaflets. Anatomically, the fibrous tricuspid annulus is indistinct and incomplete, especially at the segment corresponding to the RV “free wall” accounting for the potential dilatation in these regions whereas the “septal” segment is less prone to dilatation. The so-called annulus i.e., the hinge line of the leaflets normally circumscribes an almost oval and non-planar shape that becomes more circular as the RV dilates. Furthermore, its geometry can also be distorted e.g., in dilatation of right atrium (RA), RV as well as aortic root dilatation.

**Figure 1:**
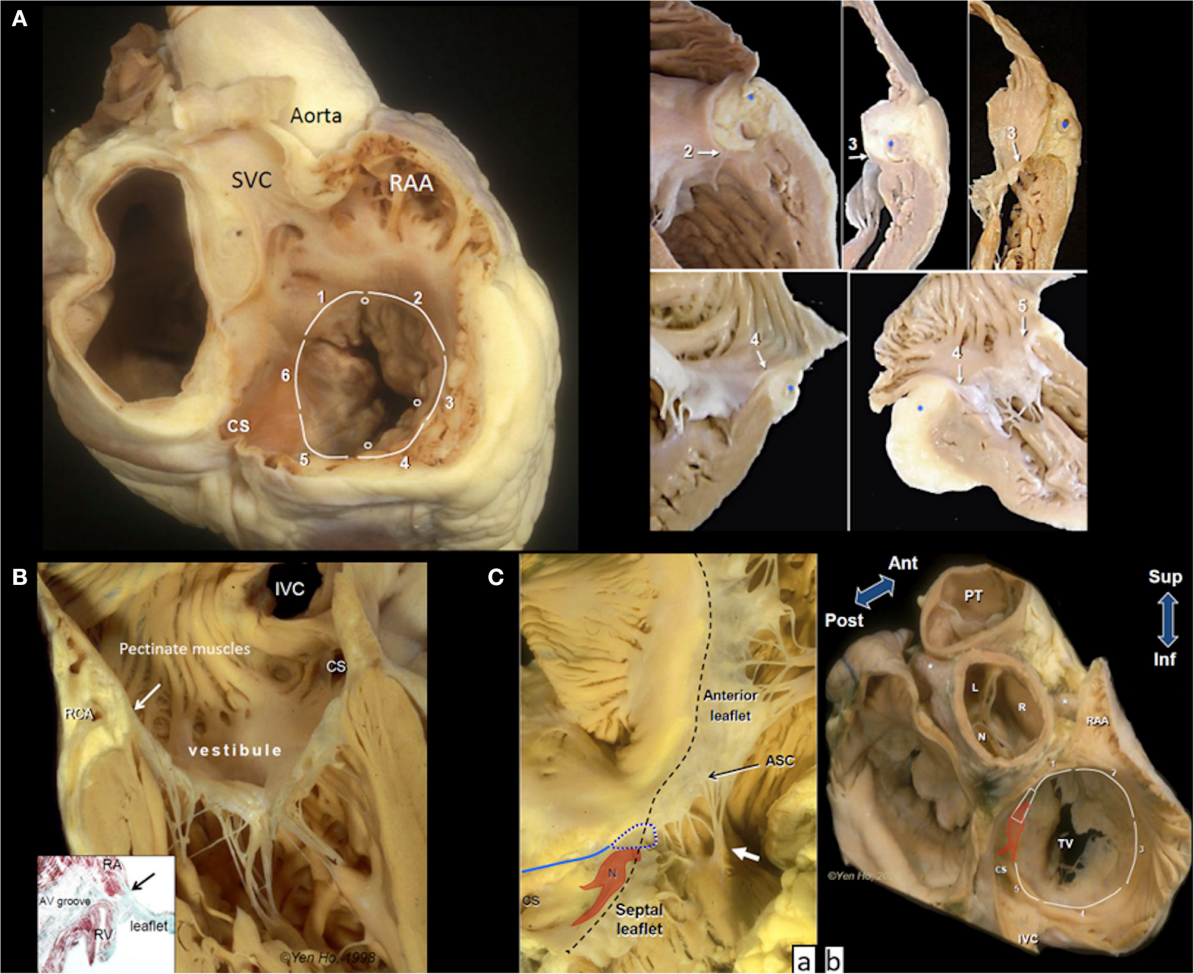
Anatomy of the tricuspid valve complex. **(A)** Main panel shows a heart cut in a plane simulating transesophageal view with the superior vena cava (SVC) at 11 O'clock position. The circles mark the leaflet commissures and the white lines trace the segments of the hinge line. Note the smooth wall vestibule of atrial wall leading to the valve orifice. The smaller panels taken from random specimens demonstrate the thinness of the vestibular wall and variations in relationship of the hinge line (arrows), depth of fat-filled atrioventricular groove, and location of right coronary artery(*) at the numbered segments. **(B)** The tricuspid valve shown in closed position with chordal insertions in the rough zone. The vestibule separates the leaflet hinge (arrow) from the pectinated part of the atrium. The right coronary artery (RCA) runs in the tissues of the atrioventricular groove (yellowish area). The inset is a histologic section cut in the same plane showing separation of atrial and ventricular myocardium (red stain). There is no robust fibrous annulus at the hinge line (arrow). Instead, fibrous tissues (green stain) from the leaflet integrate with tissues of the atrioventricular (AV) groove and adjacent atrial and ventricular walls. **(C)** (a) The atrioventricular node (N) with its two prongs of inferior extensions is located toward the apex of the nodal triangle demarcated posteriorly by the tendon of Todaro (blue line), anteriorly by the hinge line (black dashes) and inferiorly by the coronary sinus orifice (CS). In this heart there is a gap in the septal leaflet where the hinge line crosses the membranous septum (blue dotted line). The septal leaflet extends away from the septum to the antero-septal commissure (ASC) that is supported by a small papillary muscle (white arrow). This region corresponds to segment 1. (b) This basal view shows the relationship of segment 1 to the aortic through the transverse pericardial sinus. N, R, an L are the non-, right and left coronary aortic sinuses, respectively. Asterisks mark the proximal portions of the main coronary arteries. The colored shape represents the atrioventricular node and bundle penetrating through the central fibrous body (trapezoid). CS, coronary sinus orifice; IVC, inferior vena cava; RAA, tip of right atrial appendage; TV, tricuspid valve; PT, pulmonary trunk.

Imaging protocols have arbitrarily divided into 5 segments the tricuspid annulus beginning with segment 1 in the region of the antero-septal commissure and going clockwise to segment 5 at the postero-septal commissure to assess the annulus (24) (Figure 1A). In this classification the septal part of the tricuspid annulus, was separated out. Thus, the annulus along the septal leaflet is further described as segment 6. It should be noted, however, that there is significant variability in the number of leaflets (25) and lengths of the hinge line for each leaflet.

The border between RA and RV wall, the atrioventricular junction, is a wedge of tissue comprised mainly of epicardial fat intermingled with thin collagen and elastic fibers filling the atrioventricular groove (Figure 1B). This tissue is an insulating plane preventing atrial signals from directly reaching ventricular myocardium and it also contributes to the annulus of the leaflets. The right coronary artery (RCA) and its branches, and coronary veins course within the fibro-fatty tissues at variable distances and levels relative to the annulus. An anatomical study reported the distance of the RCA to the endocardial surface of the RA as furthest at segment 2 (5.3 ± 1.3 mm), closer at segment 3 (5.0 ± 2.2 mm), and closest at segment 4 (1.8 ± 0.6 mm) but did not take account of relative level from annulus (26).

The atrial wall leading to the tricuspid orifice is termed the vestibule of the RA. Its endocardial surface is smooth, separating the pectinated appendage wall from the valve orifice (Figure 1B). The portion of the vestibule corresponding to segment 5 is part of the cavo-tricuspid isthmus targeted by electrophysiologists for ablating atrial flutter. Normally the wall thickness of the vestibule is 2–3 mm, thinner in RA dilatation, and tapers considerably as it nears the annulus where it may overlap the leaflet surface (Figure 1A).

### Leaflets and Commissures

Supported by fan-shaped commissural chords, the commissures (or breach) do not reach the annulus but several millimeters of leaflet tissue remain sometimes in the form of small scallops. Although tricuspid by name, anatomically there are more commonly five leaflets (25). In addition there is also considerable variation of leaflet shape and length as well as in the presence and number of scallops (25).

All leaflets have a basal clear zone usually devoid of chordal support and a rough zone where majority of tendinous chords insert to the ventricular surface. The rough zone occupying several millimeters from the free edge corresponds to the zone of coaptation that allows the leaflets to abut together and seal the tricuspid valve orifice (Figure 1B).

The septal leaflet, as its name implies, has multiple chordal attachments directly to the ventricular septum or to multiple small papillary muscles that insert to the septum that limit excursion of its movement to close the valvular orifice (Figure 1C).

As shown in Figure 1C the inferior annulus corresponds to segment 5. The remainder of its annulus corresponds to segment 6 and part of segment 1. Although segment 6 is not deemed relevant to current transcatheter devices, it is important to understand its relationship with the cardiac conduction system, the central fibrous body, membranous septum, and the aortic root.

From the postero-septal commissure, the annulus of the septal leaflet ascends superior to be anchored to the central fibrous body which comprises of the right fibrous trigone in the region of aortic-mitral continuity and the membranous septum. This part of the annulus marks the anterior border of the triangle of Koch which is the anatomical landmark for the location of the atrioventricular node and the penetrating bundle of His sitting at its apex. The posterior border of the triangle is marked by the tendon of Todaro while the inferior border is the vestibular wall anterior to the orifice of the coronary sinus (CS) (Figure 1C). Where the annulus crosses the membranous septum superiorly to the apex of Koch's triangle, it divides the membranous septum into two components: an atrioventricular portion that separates the RA from the left ventricle (LV), and an interventricular portion that separates the two ventricles. Furthermore, the leaflet at the membranous septum may take various morphologies sometimes with accessory tissues or is partially or totally deficient thereby potentially leaving a gap in the closure line (Figure 1Ca).

Notably, where segment 6 transitions to segment 1, the septal leaflet extends laterally beyond the membranous septum at the antero-septal segment of the annulus, often appearing as a scallop that adjoins the anterior (aka antero-superior) leaflet at the commissure which is supported by the medial papillary muscle attached to the septomarginal trabeculation (septal band). Importantly, the annulus from the membranous septum to the antero-sepal commissure is related to the aortic root. On the epicardial side, the RA wall is only separated from the non- and right coronary aortic sinuses to varying extents by the transverse pericardial sinus (Figure 1Cb).

The anterior leaflet is the most extensive around the annulus, covering segments 1 through 3, and also the largest, hence contributing the most to the closure of the valvular orifice. It is usually supported by a large anterior papillary muscle that attaches to the moderator band at its insertion to the RV free wall.

Corresponding to segment 4, the posterior (or mural) leaflet is smaller, often supported by a cluster of slender papillary muscles. In some hearts the commissure between the anterior and posterior leaflets is indistinct, giving the impression of a bi-leaflet tricuspid valve while in some the leaflet is scalloped.

In segment 5, the annulus from the postero-septal commissure to continue into the septal annulus runs anterior to the orifice of the CS separated by the vestibule. The distance of the CS orifice to the annulus varies from a few millimeters to 1.5 cm or more. The vestibule may give the impression of a shelf when the orifice is located more toward the septum. This segment of the vestibule is significant in that it marks the inferior border of the anatomical landmark used for locating the atrioventricular node of the cardiac conduction system. It is the target zone for slow pathway ablation in atrioventricular nodal reentry tachycardia as it harbors the rightward inferior extension of the atrioventricular node that usually runs close to the annulus (27) (Figure 1Cb).

### Features Important for Percutaneous as Well as Surgical TV Intervention

- The RCA runs within the tissues of the atrioventricular junction at variable levels relative to the level of the leaflet annulus. Its distance from the endocardial surface of the atrium tends to be closer when it courses into the inferior segment (1.8 ± 0.6 mm) compared to its antero-lateral course (5.3+1.3 mm) (26).
- The atrioventricular node at the apex of the triangle of Koch and the conduction bundle at the border of the membranous septum with the ventricular septum. Inferior extensions of the atrioventricluar node at the annulus close to CS orifice.
- Thin RA wall at vestibule surrounding the orifice.
- Location of the aortic root.

## The Cardioband Tricuspid Valve Reconstruction System

**Figure 2:**
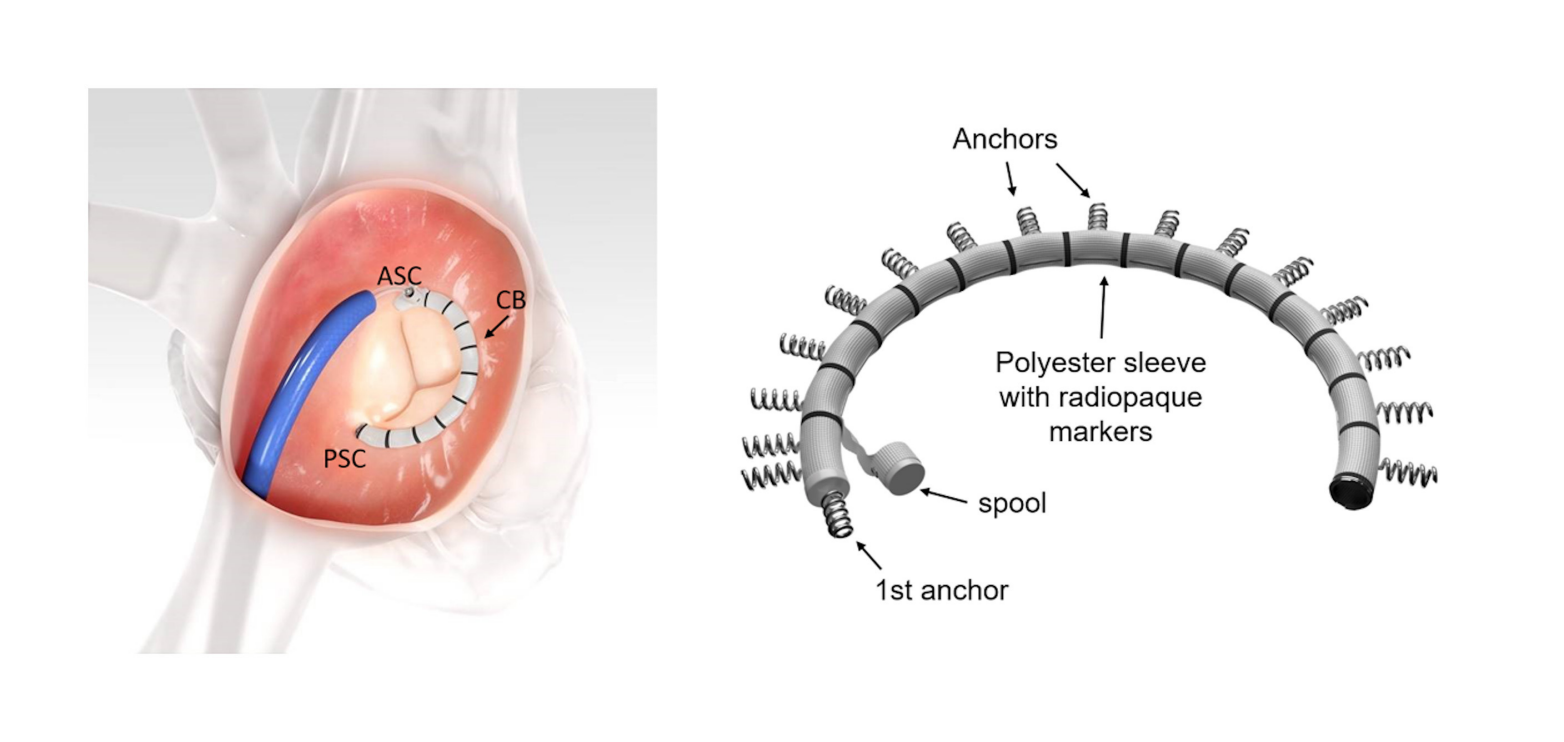
The Cardioband (CB) implant. The CB is implanted along the tricuspid annulus from the antero-septal commissural (ASC) area to the postero-septal commissural (PSC) region (left image). The specific implant features are seen on the right image. The system consists of polyester sleeve containing a pre-mounted contraction wire that is connected to an adjusting spool. On the polyester sleeve radiopaque markers are applied at a distance of 8 mm. In this example 17 anchors are present (F-band size). Note that the first anchor is at an almost 90° angle to the other anchors. Images with permission from Edwards Lifesciences, Irvine, CA, USA.

The tricuspid Cardioband (Edwards Lifesciences, Irvine, CA, USA; (Figure 2) (28) consists of a polyester sleeve containing a pre-mounted contraction wire that is connected to an adjusting spool. On the polyester sleeve radiopaque markers are applied at a distance of 8 mm. Dependent on the length of the selected band 12–17 anchors (these are screws with a length of 6 mm and a width of 2.4 mm) are deployed along the annulus under general anesthesia and 2D/3D transesophageal echocardiography (TEE) and fluoroscopic guidance starting at the antero-septal commissural area to the postero-septal commissural region. For each implanted anchor the correct positioning must be confirmed by 2D/3D TEE and fluoroscopic imaging and a pull test is used in addition to ensure secure insertion before releasing the anchor.

Once all anchors are deployed, the implant delivery system (IDS) is detached from the band and removed. A leading wire stays in place and the size adjustment tool (SAT) is inserted over the wire and connected to the spool. The implant is then contracted stepwise depending on the band size between 3.5 and 5.5 cm under beating heart conditions. TR reduction is assessed in real-time (RT) by TEE monitoring until a satisfactory result is achieved. The implant is then detached from the SAT and the entire system is removed.

### Pre-procedural Imaging

Before a procedure the exact mechanism(s) of TR, the grade of TR severity, the precise dimensions of the tricuspid annulus, the localization and extension of calcification as well as relationships of the tricuspid annulus to surrounding structures (particularly the RCA), need to be evaluated. For each anchor position it should be confirmed, that tissue quality is adequate and that there is enough space between the hinge points of the leaflets and coronary vessels to guarantee safe anchoring. As a wire in the RCA is extremely useful for procedural navigation, patency of the RCA is important, and occlusion of this vessel should be excluded prior to the procedure. Furthermore, factors that may affect imaging quality during the procedure should be addressed (see Table 1).

**Table 1:** Main imaging tasks before a Cardioband procedure and currently preferred imaging modalities.

**Main imaging task**	**Preferred imaging modalities**
Assessment of tricuspid valve pathology and definition of TR mechanism(s)	TTE, TEE
Exclusion of relevant degenerative TR components	TTE/TEE
Grading of TR severity	TTE
Assessment of annular shape, dimensions and annular calcification	MDCT
Assessment of relationships of the tricuspid annulus to surrounding structures (particularly the RCA)	MDCT
Evaluation of specific annular anchor points in relation to the hinge point of the tricuspid leaflets and coronary vessels	MDCT
Assessment of the location of pacemaker/defibrillator leads	MDCT, TTE, TEE
Evaluation of leaflet impingement by pacemaker/defibrillator leads	TTE, TEE
Evaluation of TEE imaging quality in supine position	TEE
Evaluation of factors which may cause shadowing during the procedure (e.g., presence of ASD/PFO occluders, surgical patch closure of an interatrial septal defect, mechanical mitral/aortic valves, MitraClip(s) in mitral position, pacemaker leads, lipomatous septum secundum, etc.)	TEE
Exclusion of intracardiac thrombi/masses	TEE
Evaluation of left-sided myocardial and valve disease	TTE, TEE
Evaluation of echocardiographic inclusion/exclusion criteria (see Table 4)	TTE
Definition of optimal fluoroscopic angulations for the procedure	MDCT
Evaluation of the status of the coronary arteries	Coronary angiography, MDCT angiography
Diagnosis/classification of pulmonary artery hypertension	TTE, right heart catheterization in selected patients

*TR, tricuspid regurgitation; TTE, transthoracic echocardiography; TEE, transesophageal echocardiography; MDCT, multi-detector computed tomography; RCA, right coronary artery; ASD, atrial septal defect; PFO, patent foramen ovale*.

Table 1 summarizes the main imaging views needed before the Cardioband procedure and the imaging modalities that are primarily used for this purpose.

The pre-procedural diagnostic work-up currently includes the following imaging modalities:

### Transthoracic (TTE) and Transesophageal Echocardiography (TEE)

TTE is the primary imaging modality to characterize tricuspid valve anatomy, valve lesion(s), valve function, to grade TR severity (17, 29), to evaluate RA and RV size and function (30, 31), to estimate pulmonary artery systolic pressure (32, 33) and to assess specific inclusion/exclusion criteria [inclusion and exclusion criteria used for the TRI-REPAIR study (23) are summarized in Table 2] prior to a procedure. Multiple 2D TTE views should be obtained for a comprehensive evaluation of the right heart. The advantages and limitations of each view must be known to avoid procedural errors (33, 34).

**Table 2:** Key inclusion/exclusion criteria for theTRI-REPAIR (TrIcuspid Regurgitation RePAIr With CaRdioband Transcatheter System) study (23).

**Key inclusion criteria**	**Key exclusion criteria**
**Echocardiographic inclusion criteria** • chronic secondary TR • moderate-to-severe-TR • tricuspid annular diameter ≥40 mm	**Echocardiographic exclusion criteria** • left ventricular ejection fraction ≤ 30% • systolic pulmonary pressure at rest >60 mm • aortic/mitral/pulmonic stenosis • moderate-severe aortic/mitral regurgitation • presence of pacemaker/defibrillator leads impinging a tricuspid leaflet
**Clinical inclusion criteria** • stable medical treatment • exclusion from surgery by a heart team decision • symptomatic TR	**Clinical exclusion criteria** • recent myocardial infarction or instable angina or any coronary/valve intervention ≤ 30 days before the index procedure • previous tricuspid repair/replacement

*TR, tricuspid regurgitation*.

The grading of TR is made according to current guidelines (11, 17) by using a multi-parametric approach. In this context, it is recommended to include 3D vena contracta area measurements in the quantification process (34). However, when using this method, it should be noted that due to the highly complex three-dimensional structure of the anatomical regurgitation surface area of TR, a single plane measurement may not accurately demonstrate the true anatomical orifice. Recently, an extended grading scale to better characterize TR severity prior to a transcatheter procedure has been proposed, including the new grades “massive” and “torrential” TR (35) (echocardiographic parameters indicating severe, massive and torrential TR are summarized in Table 3). Although this extended scale is not evidence-based, it may be useful to help better characterize the effect of transcatheter intervention on the TR grade. In addition, natural history studies show, that the prognosis of patients with “very severe” TR is significantly worse compared to patients with “severe” TR, thus suggesting, that any reduction of TR may result in improved outcomes and a reduction of mortality (36). However, long-term outcomes related to reduction in TR after transcatheter procedures are not currently available.

**Table 3:** Standard and newly suggested parameters to indicate severe, massive, and torrential tricuspid regurgitation (TR).

**Parameter**		**Value indicating**		
		**Severe TR**	**Massive TR**	**Torrential TR**
**Qualitative**	Valve morphology	Severe valve lesion (e.g., flail/large coaptation defect/perforation)		
	Color flow regurgitant jet	Very large central jet or eccentric wall impinging jet		
	Continuous wave Doppler signal of regurgitant jet	Dense/triangular with early peaking	Peak <2 m/s	
**Semi-quantitative**	Vena contracta width (biplane)	7–13 mm	14–20 mm	≥21 mm
	Hepatic vein flow	Systolic flow reversal		
	Tricupid inflow	E-wave dominant ≥1 m/s		
	Proximal isovelocity surface area (PISA) radius	>9 mm		
**Quantitative**	Regurgitant volume	>45 ml/beat		
	EROA	40–59 mm^2^	60–9 mm^2^	≥80 mm^2^
	Three-dimensional vena contracta area or quantitative Doppler EROA	75–94 mm^2^	95–114 mm^2^	≥115 mm^2^
	Right atrium and right ventricle	Usually dilated		

*EROA, effective regurgitant orifice area; (white, standard guideline parameters; gray, newly suggested parameters)*.

RT 3D echocardiography adds valuable information and supplements 2D echocardiography. By providing enface views of the tricuspid valve the number, size and mobility of leaflets, the attachment of the leaflets to the tricuspid annulus, leaflet coaptation, commissural opening, annular shape, and size can be evaluated during the cardiac cycle in a single view and 3D volumes can be provided (37–40).

The imaging of the tricuspid valve with TEE is generally more difficult than the mitral valve (MV). The tricuspid valve is located more anterior and thus further away from the TEE probe. Secondly, the tricuspid valve inserts more apically than the MV. Deep esophageal views often have a poorer quality because the esophagus curves away from the heart in its distal segment. Thirdly, tricuspid valve leaflets are thinner than the MV leaflets and visualization is therefore more difficult and echo drop out artifact frequently occur. Furthermore, acoustic windows are limited and fibrous/lipomatous structures in the heart as well as any prosthetic material in the left heart (e.g., prosthetic valves), at the interatrial septum (e.g., atrial septal occluders or patent foramen ovale occluders) or in the right heart (e.g., pacemaker/defibrillator leads) may cause acoustic shadowing or reverberations in the far field of the image and thus overshadow/obscure the tricuspid valve. Acoustic shadowing can usually be overcome in part by using trans-gastric windows, therefore it is important to check trans-gastric imaging quality and ensure, that the entire circumference of the annulus is visible prior to the procedure, optimally in supine position.

2D and 3D TEE imaging of the tricuspid valve is described in detail in current guidelines (41) and state-of-the art papers (39).

**Figure 3:**
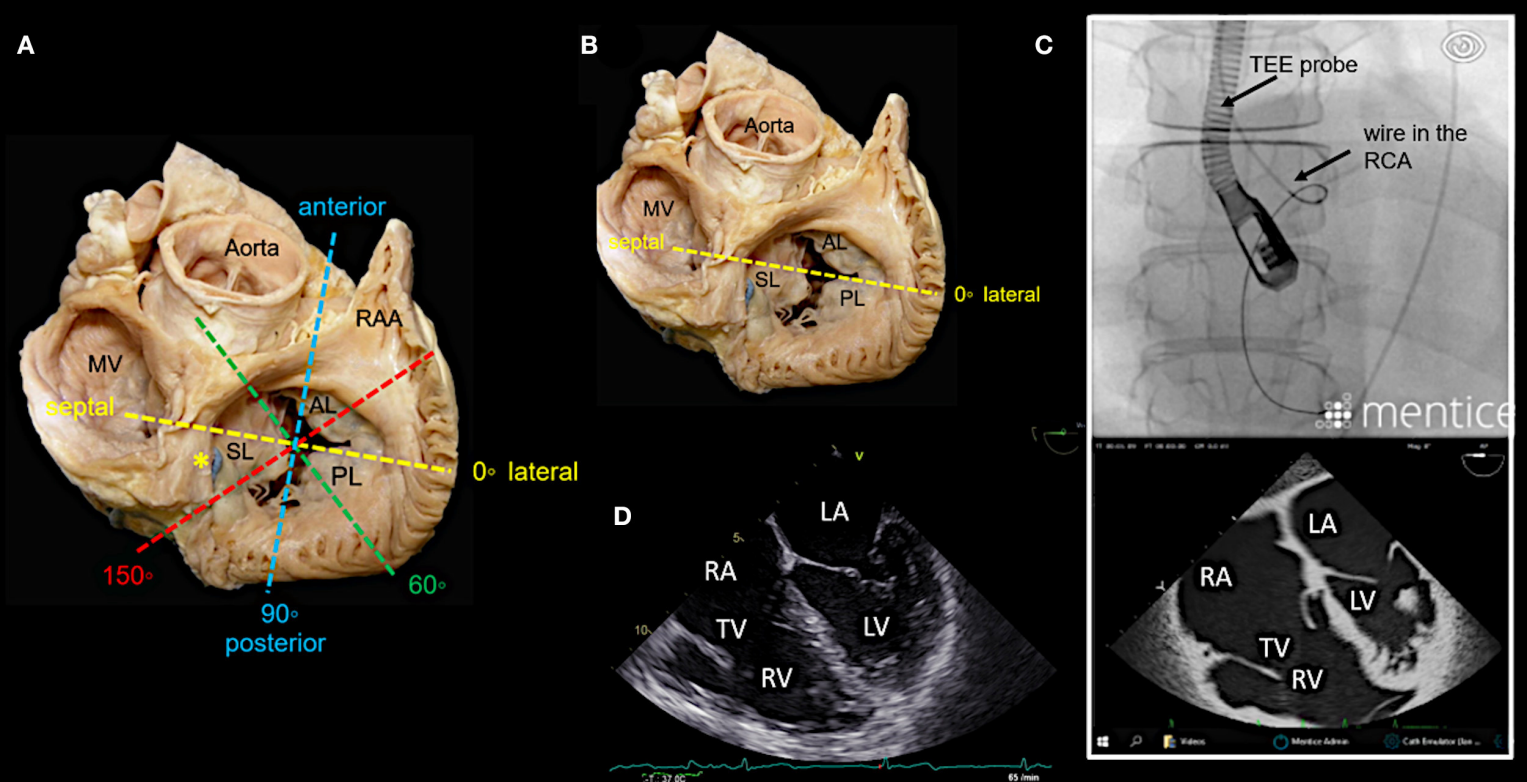
Main transesophageal echocardiographic (TEE) imaging planes for the assessment of the tricuspid valve (TV): the mid- esophageal 4-chamber view (0–10°). **(A)** The main imaging planes for assessing a TV using TEE are shown in an anatomical image of a TV with 3 leaflets. **(B)** The specific imaging plane for the mid-esophageal 4-chamber view is shown. **(C)** By using commercial simulation software (Mentice Healthy Simulation; version VIST G5; Gothenburg, Sweden) TEE and fluoroscopic views can be generated simultaneously based on MDCT data sets. The upper picture shows the necessary positioning of the TEE probe in fluoroscopy (35° RAO, 6° CRANIAL) to create a mid-esophageal 4-chamber view at 0° (see image below). **(D)** A standard 2D TEE view at 0° is shown. RA, right atrium; RV, right ventricle; RAA, right atrial appendage; RCA, right coronary artery; LA, left atrium; LV, left ventricle; MV, mitral valve; SL, septal leaflet; AL, anterior leaflet; PL, posterior leaflet; MDCT, multi-detector computed tomography; Abbreviations are also used for Figures 4–7.

**Figure 4:**
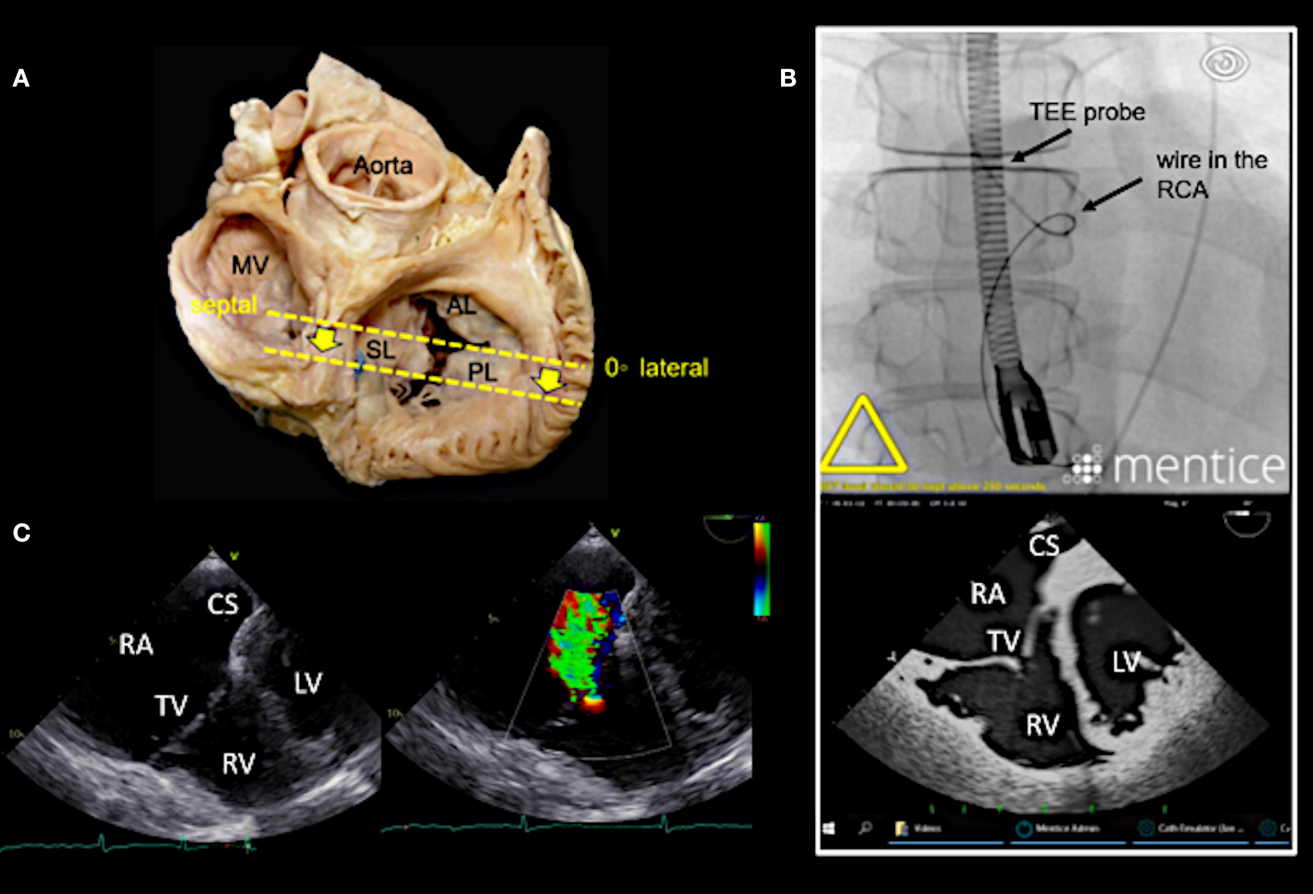
Deep esophageal view at 0–10°. **(A)** The specific imaging plane for the deep esophageal view is shown (see also Figure 3A). Starting from the mid-esophageal 4-chamber view at 0–10°, the probe is advanced deeper until the CS becomes visible. This maneuver allows for an easy evaluation of the septal leaflet. The evaluation of the posterior region of the septal and the posterior leaflets is of particular importance since the main axis of tricuspid annular dilation is in anterior-posterior direction and leaflet retraction is often pronounced in the posterior part. **(B)** The upper picture shows the necessary positioning of the TEE probe in fluoroscopy (35° RAO, 6° CRANIAL) to create the deep esophageal view at 0° (see image below). **(C)** The corresponding 2D TEE view without (left) and with (right) color Doppler is shown. CS, coronary sinus; other abbreviations see Figure 3.

**Figure 5:**
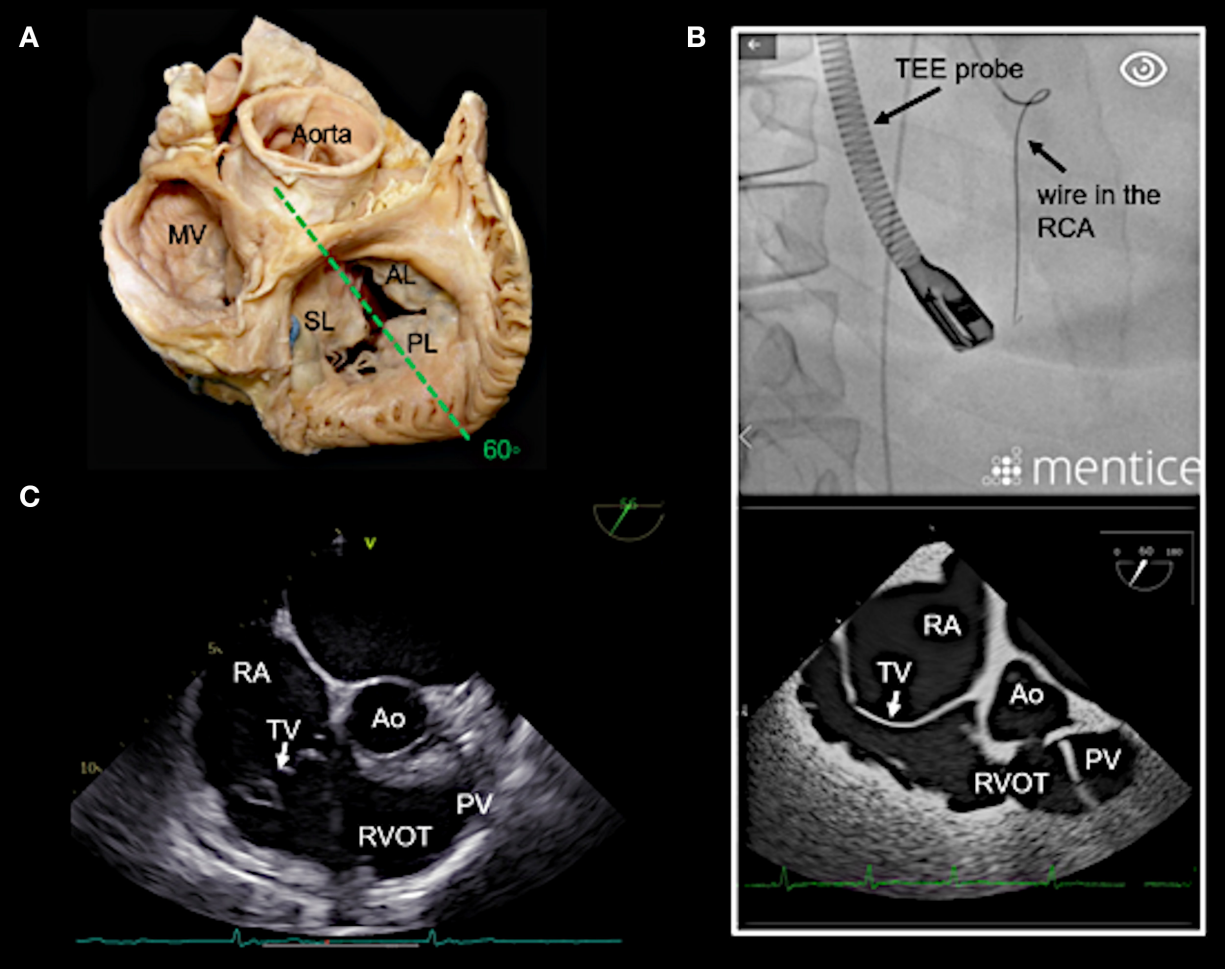
Right ventricular (RV) inflow-outflow tract view (50–70°) as seen in mid- to deep esophageal views. **(A)** The imaging plane for the RV inflow-outflow tract is found here in a deeper transesophageal view at 60° (see also Figure 3A). **(B)** The upper picture shows the positioning of the TEE probe in fluoroscopy (35° RAO, 6° CRANIAL). **(C)** A 2D TEE RV inflow-tract view is shown (slightly higher mid-esophageal position of the TEE probe). Ao, aorta; RVOT, right ventricular outflow tract; PV, pulmonary valve; other abbreviations see Figure 3.

**Figure 6:**
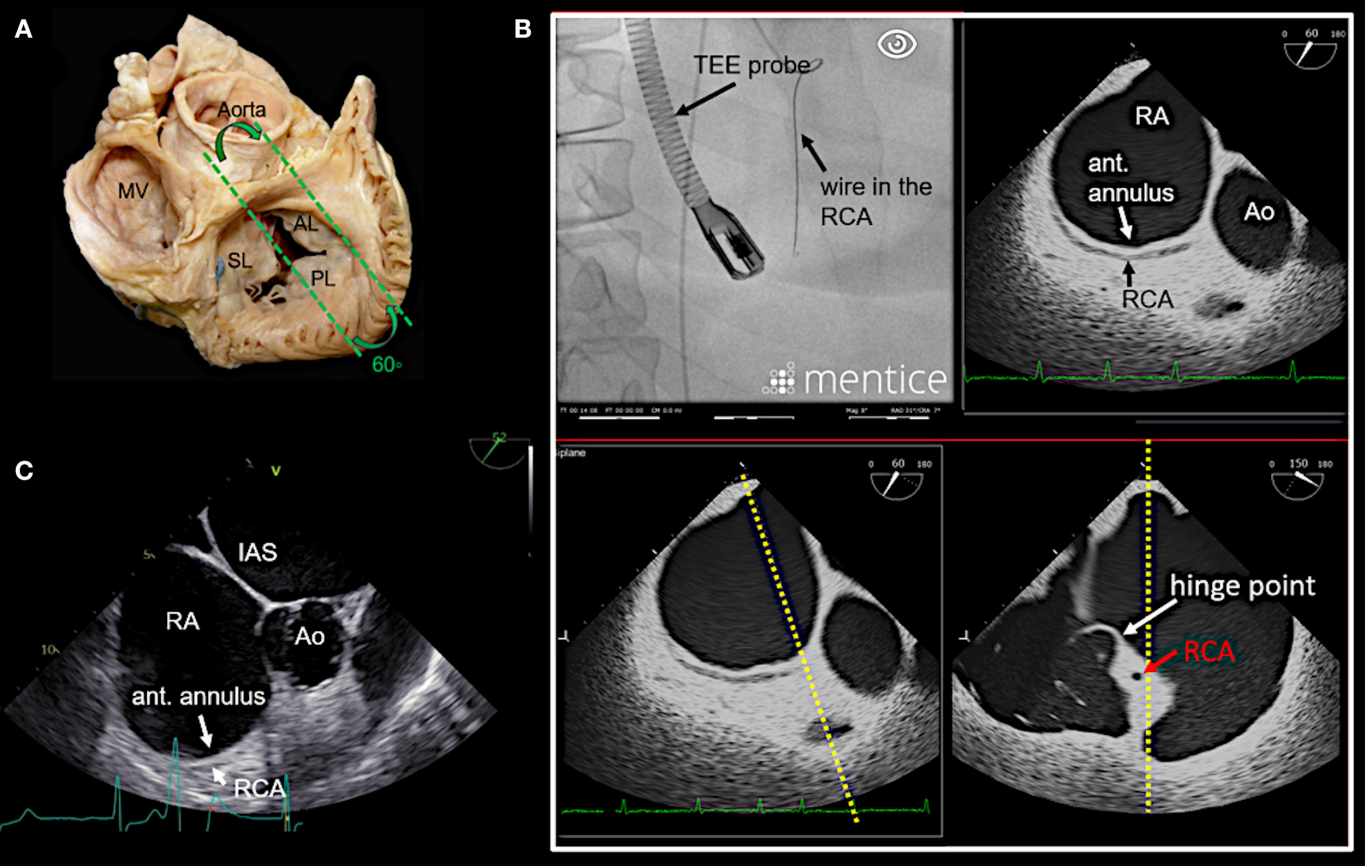
Anteriorly rotated mid- to deep- esophageal view (50–70°). **(A)** This view is achieved by rotating the probe anteriorly from the mid-esophageal or a deeper esophageal RV inflow-outflow tract view toward the anterior tricuspid annulus as shown here. **(B)** The image on the top left shows the necessary positioning of the TEE probe in fluoroscopy (35° RAO, 6° CRANIAL) to create this anteriorly rotated RV inflow-outflow tract view at 60° (image on the top right). The images below show the orthogonal long axis view (right) using x-plane imaging. During the procedure, x-plane imaging in this view can be used to identify the tip of the IC at the anterior annulus (bottom left), while in the long axis plane (right) the distance to both the RCA and the hinge point of the tricuspid leaflet can be judged. Note the course of the RCA and the proximity to annulus in this case. In **(C)** a 2D TEE view is shown. Ao, aorta; ant., anterior; IAS, interatrial septum; other abbreviations see Figure 3.

**Figure 7:**
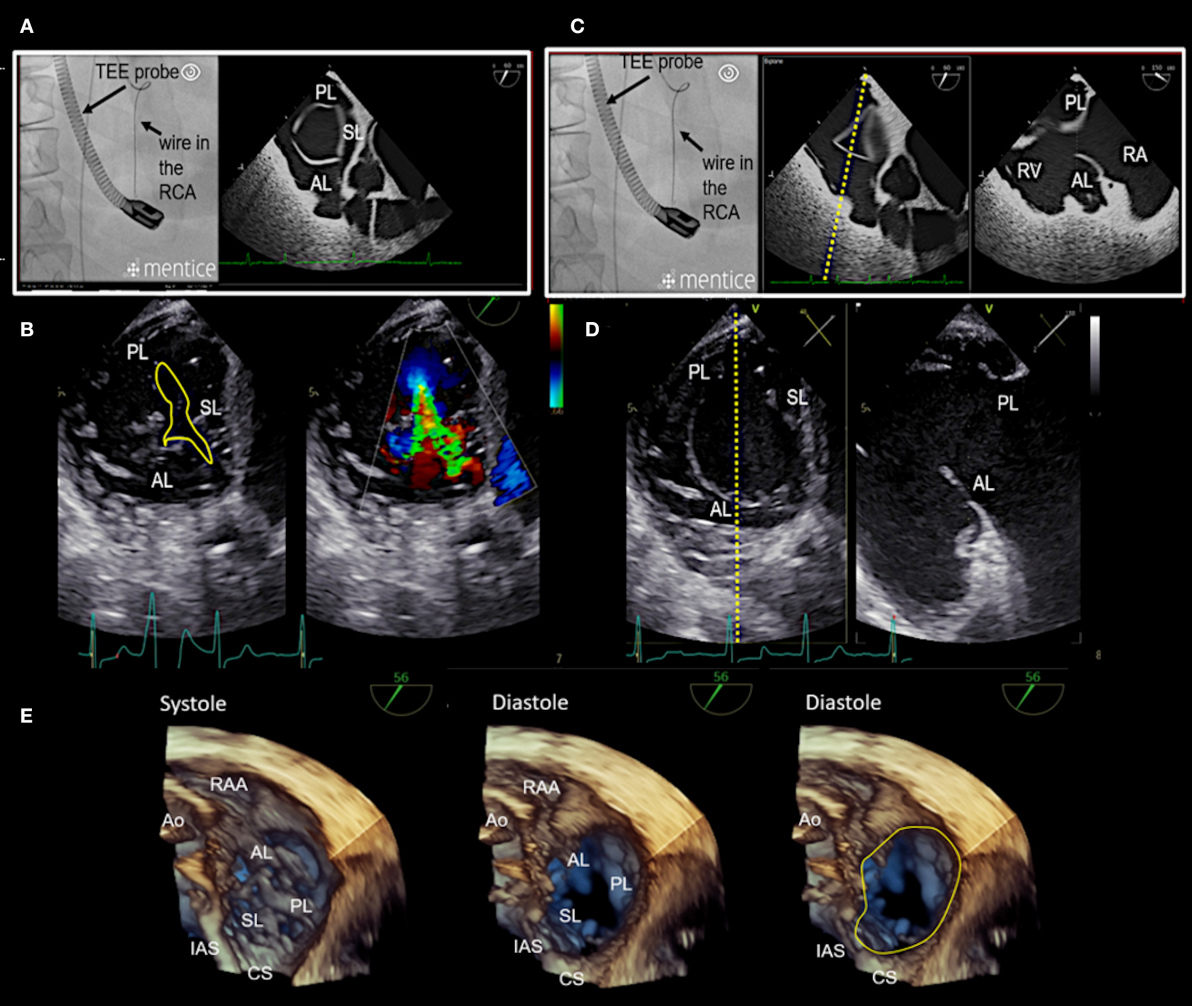
Transgastric and 3D enface views. Trans-gastric short axis views are particularly helpful, as all leaflets are usually visible. In most cases, adequate short axis views can be generated between 30 and 60°, so that the probe can be easily advanced trans-gastrally from the mid-esophageal RV inflow-outflow tract view. Additively an anterior flexion is then necessary. It should be attempted to display all commissures. **(A)** The image on the left shows the necessary positioning of the TEE probe in fluoroscopy (35° RAO, 6° CRANIAL) to create this trans-gastric short axis view at 60°. **(B)** A zoomed 2D TEE trans-gastric view obtained at 60° is shown. On the left the coaptation gap is traced (yellow line), on the right color Doppler is used to assess the origination and extension of the TR jet. **(C,D)** X-plane imaging along the anterior leaflet (images on the left) allows the evaluation of the anterior and posterior leaflet in relation to each other in long axis views (images on the right). **(E)** A three-dimensional (3D) enface view of the TV is acquired from a 60 degree view. It should be ensured, that part of the aortic valve/aortic root (Ao) and the coronary sinus (CS) is included in the 3D TEE data set. The image is than tilted and rotated so that the aorta is positioned at ~11 O‘clock and the CS at ~6 O‘clock. The entire circumference should be visible. The image on the left shows a 3D enface view in systole, in the middle a diastolic view is seen and on the right the delineation of the hinge-line is marked (yellow line) in the same view. Note the oval shape of the annulus in this case. RAA, right atrial appendage; IAS, interatrial septum; SL, septal leaflet; AL, anterior leaflet; PL, posterior leaflet; TEE, transesophageal echocardiography. Other abbreviations see Figure 3.

In our experience, the most important TEE views to assess the tricuspid valve prior to a Cardioband procedure and to guide the procedure include mid- (4-chamber) and deep esophageal views at 0–10°, a mid-esophageal RV inflow-outflow view (50–70°), trans-gastric views and 3D enface views of the tricuspid valve as shown in Figures 3–7.

### Multi-Detector Computed Tomography (MDCT)

MDCT is the preferred imaging modality to depict degenerative calcium deposition in the tricuspid annulus. This is rare in the tricuspid compared to mitral valve disease and occurs usually secondary to diffuse cardiac calcinosis, in patients with longstanding severe kidney disease and chronic dialysis and in patients with chronic inflammatory diseases (e.g., rheumatic heart disease) (42, 43). However, if present, secure anchoring in affected regions may be hindered and patients with relevant annular calcification should therefore be excluded.

**Figure 8:**
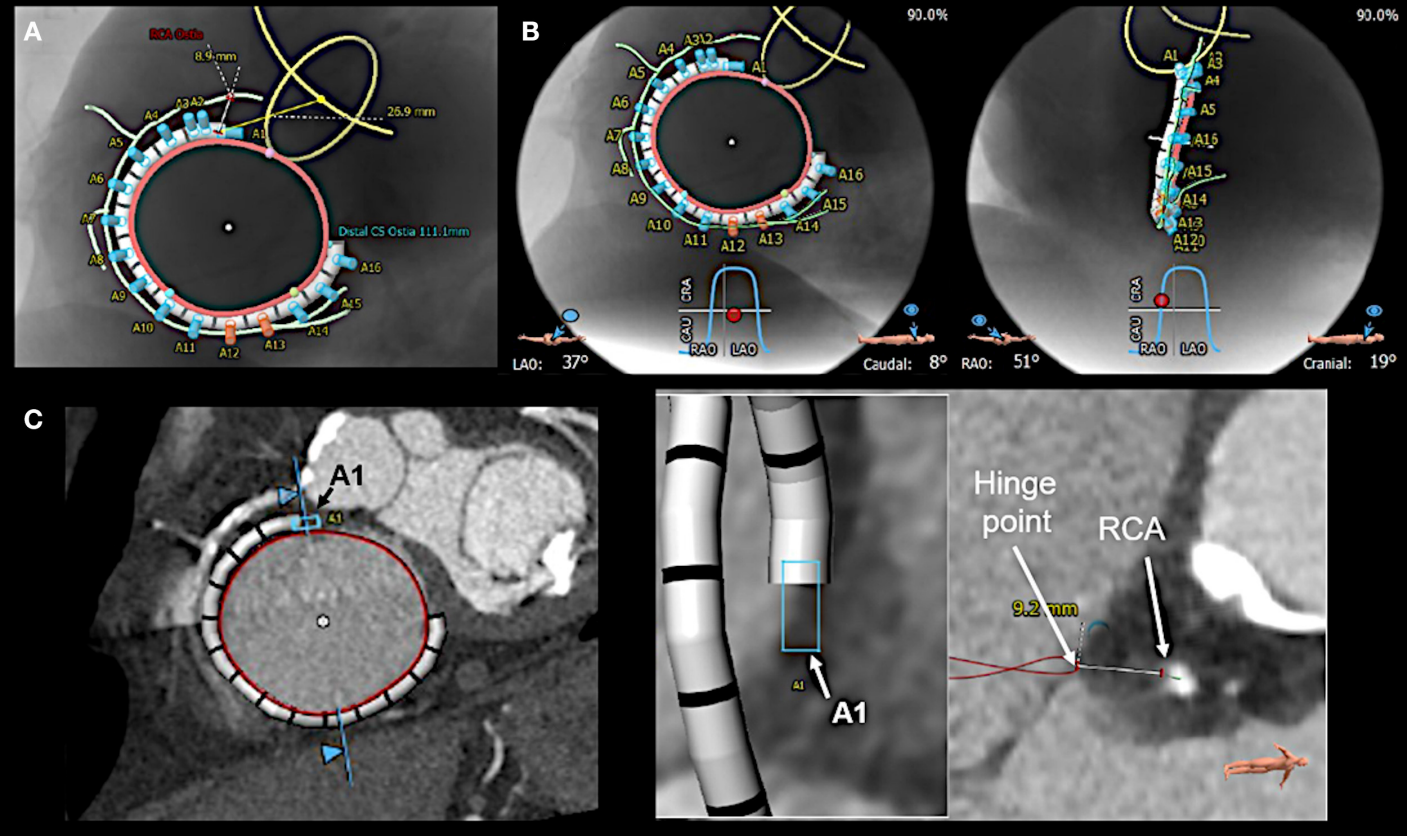
Multi-detector computed tomography (MDCT) based pre-procedural planning by using specific software (3mensio, Pie Medical Imaging BV, Bilthoven, the Netherlands). **(A)** MDCT is used to provide measurements of tricuspid annular (red circle) dimension and the distances of the anticipated anchor positions to the right coronary artery (RCA; light green line). Anchors with insufficient distance to the RCA are shown in red, the others in blue. Furthermore, the band size is determined, and the position of the first anchor point is defined as the distance from the antero-septal tricuspid annulus to the center of the aortic valve (yellow circle), which serves as a largely stable landmark. **(B)** In addition, optimal left anterior oblique (LAO; left) and right anterior oblique (RAO; right) projections for the procedure are pre-defined. **(C)** For each anticipated anchor position, the distance to the RCA and the hinge point of the leaflet is measured. Here the first anchor (A1) position is exemplarily shown.

Furthermore, MDCT is used prior to a tricuspid Cardioband procedure to measure annular dimensions, to define the position of the first anchor place, to select an appropriate band size, to evaluate the tissue quality and the distance to the hinge points of the leaflets and the RCA for each anchor region, to pre-define optimal fluoroscopic projections for a specific patient (Figure 8), and to identify the position of RV pacemaker/defibrillator leads at the level of the tricuspid valve and to detect related complications (44). However, MDCT has technical limitations in showing all tricuspid leaflet positions at a specific time frame of the cardiac cycle (45).

### Cardiac Magnetic Resonance (CMR) Imaging

CMR imaging is accurate and reproducible in the evaluation of RV morphology, size, and function and allows for the non-invasive assessment of blood flow, including stroke volume, cardiac output, pulmonary arterial distensibility and RV mass and can therefore play an important role in the decision-making process, particularly when TTE/TEE findings are ambiguous (46).

### Coronary Angiography and Right Heart Catheterization

A coronary angiography is needed prior to a procedure in order to exclude coronary artery disease. Especially the patency of the RCA should be ensured, since the placement of a coronary wire in this vessel considerably facilitates the navigation during the procedure Echocardiographic evaluation of pulmonary artery hypertension includes: a peak TR systolic velocity >2.9 m/s, an RV/LV basal diameter ratio >1, flattening of the interventricular septum in systole and/or diastole, an RV acceleration time <105 ms and/or mid-systolic notching, an early diastolic pulmonary regurgitation velocity >2.2 m/s, a pulmonary artery diameter >25 mm, an inferior cava diameter >21 mm with decreased inspiratory collapse and an end-systolic RA area >18 cm^2^.

Severe TR may cause equalization of RA and RV pressures which can result in low TR systolic velocities, leading to underestimation of pulmonary artery systolic pressures by echocardiographic Doppler evaluation (47). Consequently, a right heart catheterization should be performed to more precisely assess pulmonary pressure and the severity of hemodynamic impairment (32).

It can also result in underestimation of pulmonary artery mean pressures during right heart catheterization even in case of considerably elevated pulmonary vascular resistance. The measurement of pulmonary vascular resistance should therefore be included in the right heart catheter examination.

### Intraprocedural Imaging

The procedure is currently guided with 2D/3D TEE imaging in conjunction with fluoroscopy. Intracardiac echocardiography (ICE) has shown to be feasible for the guidance of some tricuspid procedures (48, 49) and may also be helpful during annuloplasty approaches, particularly in cases where TEE imaging is suboptimal. By positioning the ICE catheter in a steerable sheath at the level of the tricuspid valve adequate imaging to guide device positioning is achievable to our experience. The fusion of different imaging modalities, e.g., fluoroscopy and TEE imaging (EchoNavigator, Philips Healthcare, Best, the Netherlands) (39) or MDCT and TEE imaging (CT Fusion, GE Healthcare, Amersham, UK) (50) has the potential to better understand the complex anatomy of the tricuspid valve and the relationships to surrounding structures, wires, catheters and devices during procedures in future.

### Procedural Steps

**Figure 9:**
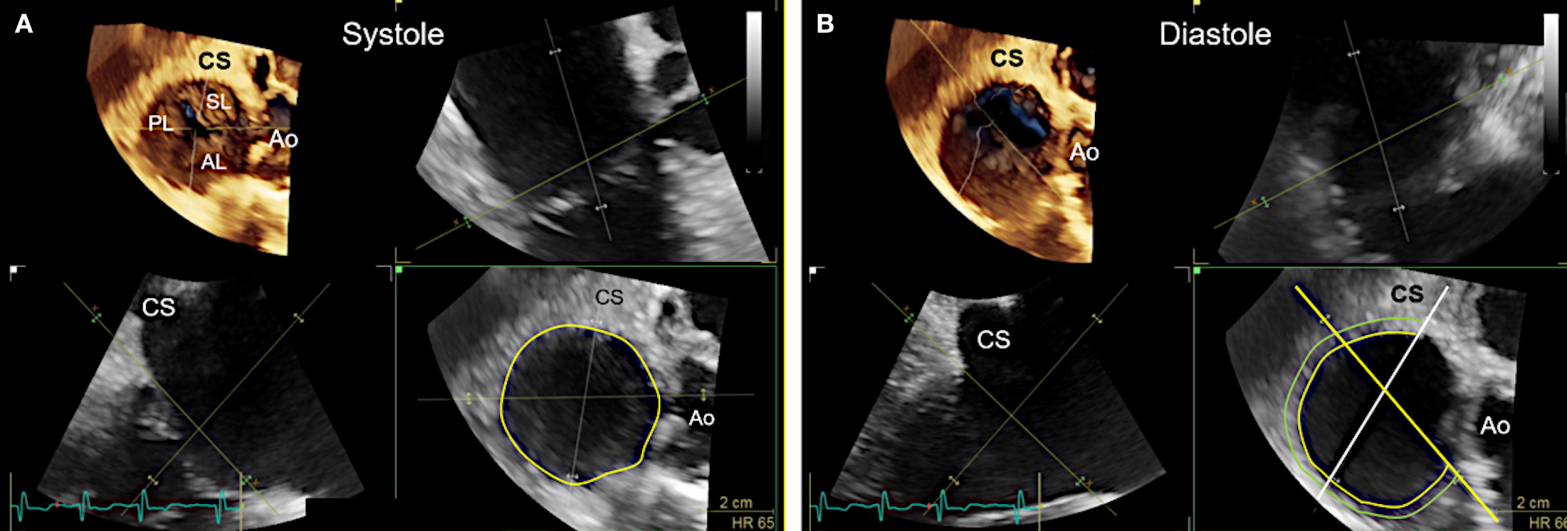
Measurement of tricuspid annular dimensions in 3D transesophageal echocardiography (TEE) by using real-time multi-planar reconstruction (RT-MPR). **(A)** The 3D data set is acquired from a mid-esophageal 60? RV inflow-outflow tract view. RT-MPR is used to measure the annular circumference (yellow circle; bottom right) after the green cropping line is adapted to the annular plane in the orthogonal views (top right and bottom left). **(B)** Measurements for band sizing should be made in diastole, when annular dimensions are largest (51). The green cropping lines are adapted to the annular plane in the same way as shown in **(A)** (top right and bottom left). In the z-plane (bottom right), the white line is then positioned at the end of the coronary sinus (CS) whereas the yellow line is positioned at the antero-septal annulus by sparing out the aorta (Ao). The hinge line (curved yellow line) is then traced in between the white and yellow line. A second line (light green) is drawn at about 4 mm away, where anchors can realistically be placed in tissue. This measurement is used to confirm the pre-selected band size which was obtained from multi-detector computed tomography imaging measurements.

Before starting the procedure 2D/3D TEE is used to re-assess annular dimensions and TR severity. This is of utmost importance as TR grade and annular dimensions are dynamic and can vary considerably in size depending on the volume status of the patient (e.g., in patients that have been fasting prior to the procedure) which may be different at the moment of the procedure compared to the point in time when MDCT derived measurements for band size selection were made. Thus, the correct band size needs to be confirmed (Figure 9). In addition, a wire [e.g., a Runthrough NS guidewire (Terumo Medical, Tokyo, Japan), or a Whisper MS guidewire (Abbott Vascular, North Chicago, IL, USA)] is positioned in the RCA to facilitate navigation during the procedure by using standard catheter techniques.

#### Introduction of the Transfemoral Steerable Sheath and the Implant Delivery System

**Figure 10:**
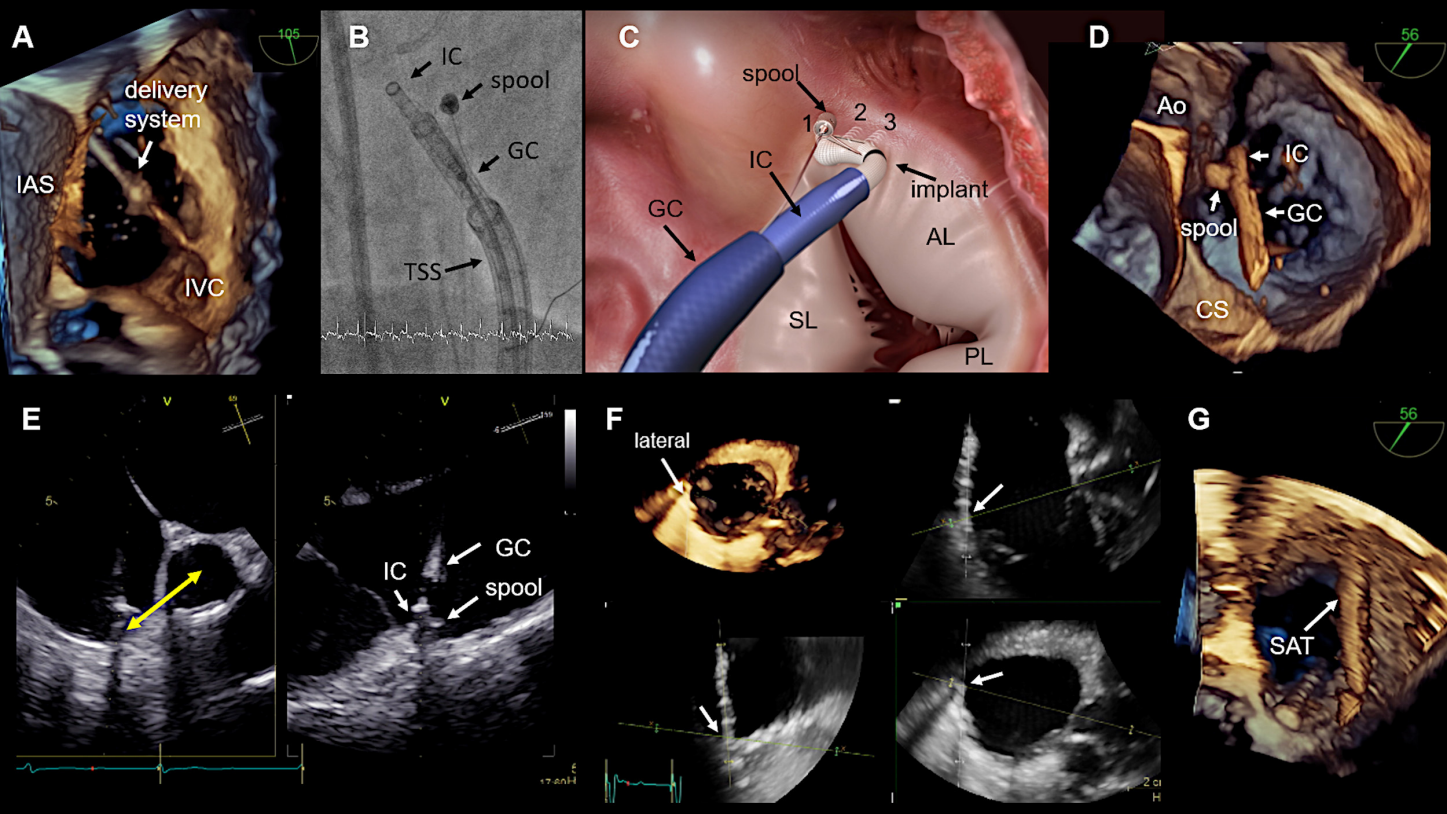
Procedural steps. **(A)** The introduction of delivery system via the IVC is seen. The tip disappears in the SVC, which is not visible. This 3D view is generated from a wide angle bi-caval view at 105°. IAS, SVC, and IVC should be integrated in the image. The image is then rotated and rotated so that the IAS is located on the left (medial) side. **(B)** The individual catheter components as seen in fluoroscopy. **(C)** In this schematic the catheter components, the implant and the first three implanted anchors (1,2,3) in the antero-septal commissural area are shown. **(D)** The navigation of the delivery system toward the first anchor position is seen in a 3D TEE enface view. **(E)** The measurement from the IC tip at the anterior annulus to the middle of the aorta which confirms a correct 1st anchor position is seen on the left side. X-plane imaging is used to evaluate the distance to the hinge point of the leaflet (right). Lateral positioning of the spool must be avoided, otherwise the contraction wire may be damaged during anchoring. **(F)** The use of real-time multi-planar reconstruction to confirm adequate distance from the IC tip to the hinge point of the leaflet is demonstrated for an anchor in lateral position. **(G)** In this 3D TEE enface view the SAT is seen which is connected to the spool. IAS, interatrial septum; IVC, inferior cava; IC, implantation catheter; GC, guide catheter; TSS, transfemoral steerable sheath; SL, septal leaflet; AL, anterior leaflet; PL, posterior leaflet; CS, coronary sinus; SAT, size adjustment tool; TEE, transesophageal echocardiography. (Image B with permission from Edwards Lifesciences, Irvine, CA, USA).

In a first step, the transfemoral steerable sheath (TSS) with a dilator is introduced into the RA or directly into the superior caval vein (SVC) under fluoroscopic and TEE guidance by using a transfemoral venous access. In the next step, the implant delivery system (IDS) consisting of the steerable Guide catheter (GC) and the implantation catheter (IC; can only be retracted or advanced and is not steerable) is inserted and positioned in the SVC. These steps are best monitored by using 2D TEE bicaval views with x-plane imaging or alternatively 3D TEE views obtained from a bicaval view (Figure 10A). The entire system is then retracted until TEE imaging confirms, that the tip of the IDS is no longer in the SVC and the delivery system is then navigated downwards toward the antero-septal commissural area of the tricuspid annulus to reach the first anchor position.

#### Device Implantation

In general, 3D TEE enface views of the tricuspid valve are used to navigate the delivery system around the tricuspid annulus to the target region. Once an anchor point is reached, x-plane imaging or RT multi-planar reconstruction (RT-MPR) of a 3D data set is subsequently used to confirm contact of the IC tip to the annulus, adequate distance of the IC tip to the hinge point of the leaflets and coronary vessels, an adequate angulation of the delivery system in relation to the annular plane and satisfactory tissue quality for anchoring. After the correct position is confirmed by TEE and fluoroscopy, the anchor is screwed in and subsequently a tug- test is performed to ensure stable anchorage. During the tug- test attention must be paid to the annular tissue movement at the anchor point (visible in TEE), alternatively a relative movement between the GC and IC can be considered as an indirect sign of secure anchoring (visible in TEE and fluoroscopy). If the anchor position is satisfactory the anchor is released and by releasing the band the next anchor position is targeted. All further anchors are implanted in the same way along the tricuspid annulus. The procedural steps are illustrated in Figure 10 and specific anatomical and imaging considerations for anchor placement in the different annular regions are summarized in Table 4.

**Table 4:** Specific anatomical and imaging considerations for anchoring in the different regions of the tricuspid annulus.

**Anchor region**	Segment^*^	**Anatomical considerations**	**Imaging considerations**
Antero-septal commissural (ASC) area	1	-Proximity of the aortic root to the annulus	-The 1st anchor point should be as close as possible to the ASC to achieve maximum efficacy after contraction -The distance from 1st anchor point to the middle of the aorta needs to be confirmed (see Figure 10E) -For the 1st anchor meticulous care is warranted during anchor placement in patients with aortic root dilation/deterioration -Shadowing is frequent in this region (caused by a sclerotic aortic valve/root or an aortic prosthesis); transgastric views are frequently needed -A lateral position of the spool must be avoided, otherwise the contraction wire can be damaged during anchoring
Along the hingeline of the anterior leaflet	2	-Proximity of the RAA orifice to the annulus-RCA is usually furthest away in this region	-The RAA may originate close to the annulus and maneuvers within the RAA body should be avoided by TEE monitoring -TEE x-plane imaging is usually sufficient for anchor placement in this region
Antero-posterior commissural (APC) area	3	-RCA may be close to the annulus-Presence of chordal structures	-The annulus is usually less well defined in TEE in the APC region -Chordal structures may be misinterpreted as leaflets RT-MPR of 3D TEE datasets is frequently helpful in this region
Along the hingeline of the posterior leaflet	4	-RCA is usually closest to the annulus in this region	-Most difficult part to image as it is the most inferior -A “hooking” of the implant system is needed to reach anchor points; thus imaging of the implant system becomes more difficult and “pull-tests” are more difficult to judge -RT-MPR of 3D TEE datasets is frequently helpful in this region
Postero-septal commissural (PSC) area	5	-Proximity of the CS to the annulus (varying distance)-Presence of chordal structures-Rightward inferior extension of the atrioventricular node close to the hinge line may be present	-Optimally the band should end anterior of the CS orifice for maximum efficacy after contraction -As the major axis of annular dilation is anterior-posterior leaflet retraction is usually more pronounced in the posterior part. In case of asymmetrical tethering of the posterior part of the septal leaflet and the posterior leaflet residual TR may persist after contraction -Chordal structures may be misinterpreted as leaflets -A “hooking” of the implant system is needed to reach anchor points, thus imaging of the implant system becomes more difficult and pull test are more difficult to judge -RT-MPR of 3D TEE datasets is frequently helpful in this region

* *See Figure 1A*.

*ASC, antero-septal commissure; RAA, right atrial appendage; RCA, right coronary artery; TEE, transesophageal echocardiography; TR, tricuspid regurgitation; RT-MPR, real-time multi-planar reconstruction*.

#### Contraction of the Implant

**Figure 11:**
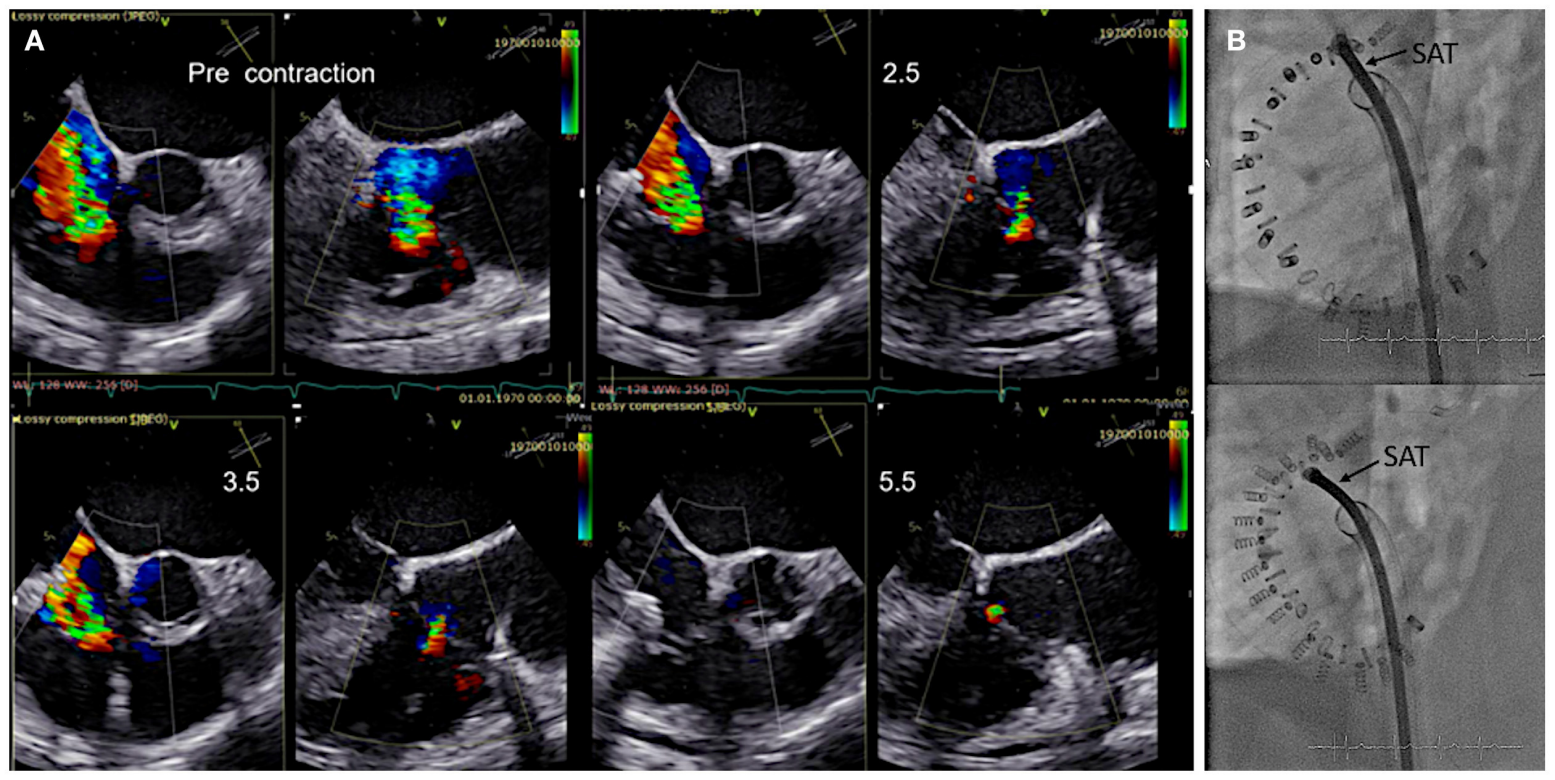
Monitoring during step-wise contraction of the implant. **(A)** Evaluation of the reduction of tricuspid regurgitation (TR) under gradual increasing contraction (2.5, 3.5, and 5.5 cm) are shown in x-plane imaging with color Doppler (TEE 60? left/150? right). Under maximum contraction (5.5 cm; bottom right) only trivial residual TR is visible. **(B)** The implant size and shape is seen in fluoroscopy before (upper image) and after (lower image) contraction. SAT, size adjustment tool.

Once all anchors are implanted the IDS is retrieved and the SAT is inserted over the wire and connected to the spool. The contraction is performed step-wise and the effect on the TR grade and annular dimensions is monitored by TEE preferably by using x-plane imaging with color Doppler of the TR jet under beating heart conditions as shown in Figure 11 until a satisfactory result or the maximum possible contraction for the selected band size is reached.

#### Assessment of Final Result

Before the device is detached from the SAT annular dimensions and the grade of TR are re-assessed in the same way as before and a coronary angiography is performed to confirm unlimited flow in the RCA. If needed, the band can be partially or completely released prior to detachment. If the result is satisfactory, the implant is detached from the SAT and the entire system is retrieved through the femoral vein.

#### Detection of Complications

Complications may occur at any time point during the procedure. These include the formation of thrombi either in the cardiac chambers or attached to wires/catheters/devices, the occurrence of pericardial effusion/tamponade or a peri-device leak, anchor disengagement, injury of the RCA or cardiac veins including the CS, injury of tricuspid leaflets, arrhythmias, particularly higher grades of AV blocks, or RA/RV/defibrillator lead dislodgement. Thus, continuous, detailed and active TEE monitoring, as well as ECG and hemodynamic monitoring is required to immediately detect any of these complications.

#### Specific Considerations in Patients With Pacemaker/Defibrillator Leads

If pacemaker/defibrillator leads are present, certain questions must be answered:

- How many leads are present?
- Is one of the leads relevantly involved in the mechanism of TR?
  ∘ If yes, the primary consideration should be to revise the lead to improve the TR
- If a RA lead is present, where is the tip located?
  ∘ Most RA leads are located in the anterior region close to the right atrial appendage. In this case it is usually easy to navigate the delivery system to the lateral side of the lead. If the RA lead is located at the lateral wall, it is necessary to insert the delivery system inferior to the RA lead to avoid any interference with the delivery system
- Where are the RV/defibrillator leads located at the level of the Tricuspid valve and are the leads mobile or attached to the annulus/to a tricuspid leaflet/to the RA lead?
  ∘ In general, the delivery system should be navigated to the lateral/anterior side of the RV/defibrillator leads to avoid any interference
  ∘ If a lead is mobile it is usually possible to push the leads toward the poster-septal commissure and to avoid any interference with the delivery system.
  ∘ If it is not possible to navigate the system to the lateral/anterior side of the RV/defibrillator leads or if the lead(s) are attached to the annulus, to each other or to leaflets, a careful analysis should be done, to decide whether the procedure can be performed without endangering the pacemaker/defibrillator leads.
- Are there any thrombi attached to the leads?
  ∘ Mobile thrombi attached to pacemaker leads are frequent and occur in up to 30% of patients undergoing ablation therapy (52). If a patent foramen ovale is present in addition, a higher risk of stroke may be present (53).

## Conclusion

Secondary TR is the most common etiology occurring in about 90% of patients with severe TR. Therefore, interventional approaches that target the mechanistic causes of secondary TR in the form of annulus reduction are particularly important. In order to perform an annuloplasty procedure effectively and safely, a good understanding of the anatomy of the complex tricuspid valve apparatus and especially of the annulus in relation to the important neighboring structures such as the aortic root, the RCA, the electrical pathways and the CS is fundamental. In addition, detailed knowledge of the device itself, the delivery system, its maneuverability and the individual procedural steps is required. Furthermore, the use of multi-modality imaging is extremely important. For each step of the procedure, the imaging modality and the optimal imaging planes that are best suited to provide the necessary information are essential to guide the individual procedural step and lead to a safe, successful, and effective procedure.

## Author Contributions

All authors listed have made a substantial, direct and intellectual contribution to the work, and approved it for publication.

## Conflict of Interest

NW reports being a consultant and proctor for Edwards Lifesciences and a consultant for Bioventrix Inc. MA reports being a consultant for Edwards Lifesciences and Abbott Vascular and MS reports being a lecturer and proctor for Abbott Vascular, Boston Scientific, Philips Healthcare and Bioventrix Inc. The remaining authors declare that the research was conducted in the absence of any commercial or financial relationships that could be construed as a potential conflict of interest.

## Abbreviations

2D: two-dimensional
3D: three-dimensional
ASC: antero-septal commissure
ASD: atrial septal defect
CS: coronary sinus
CMR: cardiac magnetic resonance
ECG: electrocardiogram
EROA: effective regurgitant orifice area
FAC: fractional area change
GC: guide catheter
IC: implant catheter
ICE: intracardiac echocardiography
IDS: implant delivery system
LV: left ventricle
MDCT: multi-detector computed tomography
MPR: multi-planar reconstruction
MV: mitral valve
PFO: patent foramen ovale
PSC: postero-septal commissure
RA: right atrium
RCA: right coronary artery
RT: real-time
RV: right ventricle
SAT: size adjustment tool
SVC: superior caval vein
TEE: transesophageal echocardiography
TTE: transthoracic echocardiography.
